# Supplementary material for: The HCV-Dependent Inhibition of Nrf1/ARE-Mediated Gene Expression Favours Viral Morphogenesis
Source: Viruses. 2025 Jul 28;17(8):1052. doi: 10.3390/v17081052 (PMC12390641; doi:10.3390/v17081052)
Supplement: Supplementary file 1 [file viruses-17-01052-s001.zip › viruses-3727021-supplementary.pdf]

**The HCV-dependent inhibitionof Nrf1/ARE-mediated gene expression favours viral morphogenesis**

**Olga Szostek [1], Patrycja Schorsch [1,2], Daniela Bender [1], Mirco Glitscher [1] and Eberhard Hildt [1,3,*]**

[1] Paul-Ehrlich-Institute, Research Group, 63225, Langen, Germany
[2] Independent Researcher
[3] Universität Potsdam, Hasso-Plattner-Institut, Digital Health Cluster, 14482, Potsdam, Germany
* Correspondence: eberhard.hildt@pei.de

## Supplementary Information

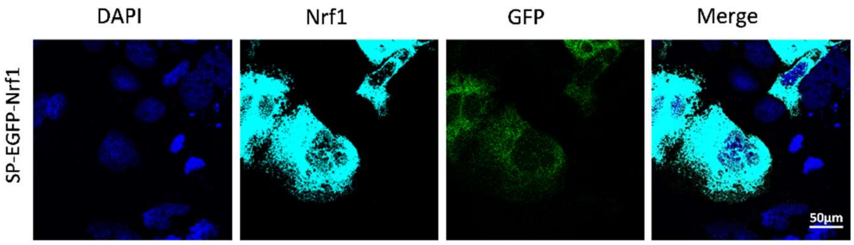


**Figure S1** Specificity of anti-Nrf1 antibody that binds N-terminally to the protein confirmed by expressing Nrf1 N-terminally tagged with EGFP. CLSM analysis. Huh7.5 cells were transfected with plasmid encoding full length Nrf1 tagged N-terminally with EGFP (green). Signal peptide (SP) targeting the protein to ER enables its proper localization. The cells were fixed with 4% PFA. For the full length Nrf1 detection a specific antibody binding to the N-terminal part of the protein was used (cyan). Nuclei were stained with DAPI. Scale bar, 50µM.

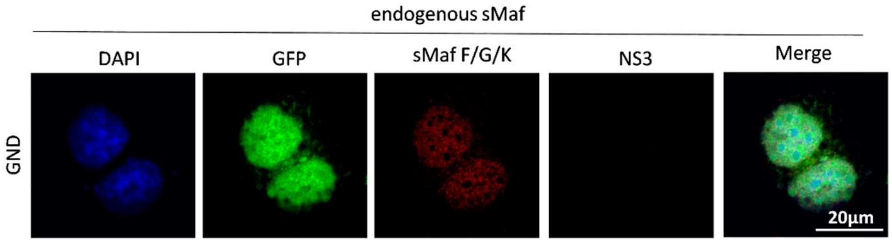


**Figure S2** sMaf proteins are localized in the nucleus in GND cells. CLSM analysis. Huh7.5 control cells (GND) were transfected with a plasmid expressing EGFP protein (green). For detection of endogenous sMaf proteins, specific antibody was used (red). Lack of HCV specific proteins is confirmed by no signal detected after NS3 was stained using specific antibody. Nuclei were stained using DAPI (blue). Scale bar, 20 µm. Images are representative of 3 biological replicates.
